# Fear of falling in community-dwelling older adults: A cause of falls, a consequence, or both?

**DOI:** 10.1371/journal.pone.0194967

**Published:** 2018-03-29

**Authors:** Ana Lavedán, Maria Viladrosa, Pilar Jürschik, Teresa Botigué, Carmen Nuín, Olga Masot, Raquel Lavedán

**Affiliations:** 1 Department of Nursing and Physiotherapy, University of Lleida, Lleida, Spain; 2 University Hospital Arnau de Vilanova, Lleida, Spain; 3 University Clinical Hospital Lozano Blesa, Zaragoza, Spain; Ludwig-Maximilians-Universitat Munchen, GERMANY

## Abstract

**Background:**

Despite the number of studies that have tried to demonstrate that there is an association between previous falls and the fear of falling, the relationship between these two variables remains a matter of controversy.

**Objectives:**

Our objective was to investigate whether the fear of falling is a cause of falls, a consequence, or both in community-dwelling adults aged ≥ 75 years old.

**Methods:**

A descriptive, longitudinal, prospective study was performed. A total of 640 individuals were interviewed between 2009 and 2011. Sociodemographic data, health status, history of falls and fear of falling were assessed at baseline and at 24 months.

**Results:**

The prevalence of falls at baseline was 25% as opposed to 35.2% at 24 months. The prevalence of the fear of falling was 41.5% at baseline. Logistic regression analysis showed a significant association between a history of falls and the fear of falling. Other factors associated with the fear of falling were female gender, comorbidity, depressive symptoms, and disability. In total, 41.7% of the subjects who had reported a fear of falling at baseline had suffered at least one fall 24 months later. Unadjusted Cox regression analysis revealed that the fear of falling was a risk factor for falls. According to the final model adjusted for other covariates, the only reliable predictor was female gender. The Cox model stratified by gender failed to show a crude association between fear of falling and falls.

**Conclusion:**

A previous history of falls in the previous year was a good predictor of the fear of falling; but the fear of falling was a predictor of falls during follow-up only in the unadjusted model, pointing to strong gender turns out as an effect modifier of the association of FOF and subsequent falls. Nursing staff working in elderly care should not only routinely assess patients’ previous history of falls, but also evaluate their fear of falling and its associated factors.

## Introduction

In recent decades, a number of studies have been performed to investigate accidental falls in older adults, who may subsequently develop a fear of falling (FOF) [1]. A fear of falling is defined as a cautious concern with falling which ultimately results in a restriction of activities associated with daily life [2]. FOF became recognized as a specific health problem when Murphy & Isaacs [3] described "post-fall syndrome" and identified FOF as a primary symptom of this syndrome. At present, FOF forms part of routine assessment and care for older adults.

This syndrome is common among community-dwelling older adults. However, there is wide variability in the prevalence rates reported, which range from 21% to 85% [4]; this is probably due to the lack of consensus on its operationalization and inconsistency in the variables assessed.

It is now widely accepted that FOF is multifactorial [5–7] and multidimensional [8]. There is evidence that FOF is more common among women [4,9–13] and older subjects [14,15] and that it is associated with impaired functional capacity [16,17], decreased cognitive capacity [16] and depression [18].

Despite the number of studies that have tried to demonstrate a relationship between FOF and previous falls [6,19,20], there is also evidence to suggest that FOF is not always a consequence of a previous fall [21,22]. Some studies have suggested that FOF could be considered a risk factor for falls [12,23], whereas others studies have raised doubts about such a relationship [24–28]. Allali et al. [26] found that FOF was associated with future falls in older adults without dementia; yet, FOF was only a predictor of falls in individuals with postural instability/gait difficulty. Likewise, Basaran et al. [27] and Palagyi et al. [28] found that FOF was a predictor of falls when the analysis was adjusted for age.

The purpose of this study was to shed light on controversies relating to FOF and to determine the correlation between FOF and falls. The primary objective of this study was to investigate whether FOF is a cause of falls, a consequence, or both, and to determine its risk factors in community-dwelling adults aged ≥ 75 years old.

## Methods

### Study design, setting and subjects

This study is part of a larger longitudinal study: ‘‘Assessing the frailty process in older adults in Lleida”, whose aim was to assess and analyze a follow-up survey conducted on an elderly population from Lleida. Data were collected using the *Encuesta de Fragilidad de las personas mayores de Lleida* (FRALLE—Survey on Fragility in older people in Lleida) [29].

The subjects were first stratified by primary healthcare centers (n = 7) and then selected at random. The sample composed of community-dwelling men and women aged ≥ 75 years old from Lleida, Spain. Lleida is a city located in northwest Spain with a population of 145,234, of whom 11,846 are ≥ 75 years old.

The inclusion criteria were male and female gender, age ≥75 years old, living at home, coverage by the public health system, and signed informed consent. The exclusion criteria were residential care, terminal illness and cognitive impairment (Pfeiffer ≥ 3) [30] without accompanying carers.

### Data collection

Face-to-face interviews were conducted by nurses trained in data collection who used the FRALLE survey which incorporated all of the variables considered in the study. Interviews were conducted at each subject's primary care center or at the participant’s home if they were disabled. Data collection was performed between 2009 and 2011. When randomly selected individuals either could not be found or did not want to participate in the study, they were replaced by others in order to meet the target sample size; this was the case with 22% of those initially selected. The second-wave interview was conducted via telephone, two years later.

### Measurements

A series of data (listed below) were collected at baseline. All of these measurements had previously shown with good levels of reliability and validity for elderly people.

#### Fear of falling

FOF was investigated using a dichotomous yes/no question: *“Are you afraid of falling*?*”* [31]. This question has been extensively used in previous studies involving community-dwelling older adults and its reliability [16,32] and criterion validity have been appropriately demonstrated [33,34].

#### Falls

Falls were operationalized as the consequence of an event which had resulted in a person inadvertently coming to rest on the ground. The number of previous falls was established via the question: *“Have you had any falls in the last year*?*"* An affirmative answer was considered to indicate a previous history of falls. If there were 3 or more errors in the Pfeiffer test, indicating significant cognitive impairment, the answer had to be provided by a PROXY. We also registered the number of falls; this information was subsequently analyzed in two different categories (1 fall; 2 or more falls).

#### Sociodemographic data

The variables recorded were age, gender, civil status, live alone or accompanied and monthly income.

#### Cognitive impairment

This was assessed using the Pfeiffer test [30]. This test consisted of 10 questions and included a correction factor for the educational level of the respondent. Errors were counted and a score of ≥ 3 was taken to indicate cognitive impairment.

#### Comorbidity

This was determined using Charlson’s comorbidity index [35]. This index is based on 19 factors associated with comorbidity. Each factor was assigned a value according to the relative risk of death, giving a maximum score of 37. A score of ≥ 3 corresponded to comorbidity.

#### Symptoms of depression

These were assessed using the Center for Epidemiologic Studies Depression Scale (CES-D) [36]. The CES-D involves the use of a 20-item questionnaire to assess the presence of symptoms of depression. A score of ≥ 16 indicates that the subject has symptoms of depression.

#### Functional capacity

Functional capacity was measured by the Katz Index of Independence in activities involved in daily living (ADL) [37] and the Lawton and Brody Scale of instrumental activities of daily living (IADL) [38]. The Katz Index for ADL was obtained using a questionnaire that assesses six basic daily life activities. The respondents were given a score of 0 if they were able to perform an activity autonomously and a score of 1 if they required assistance or could not perform the activity at all. The Katz Index has consistently demonstrated its utility for evaluating functional status in the elderly and as a predictor of adaptive capacity in the context of community residences and congregate living facilities [39]. Its interrater reliability is 0.95 and its coefficient of replicability is 0.94–0.97 [40]. Its sensitivity and ability to detect small changes in the functional status of participants was judged acceptable [38].

The Lawton and Brody Scale for IADL was obtained after evaluating eight instrumental activities. The final score ranges from 0 (maximum dependence) to 8 (total independence). The Lawton IADL is a generic scale which was developed for community-resident older adults [38]. The Lawton IADL scores were obtained with Cronbach alpha settings of 0.91 and 0.78 [41,42]. The results of Vergara et al. [43] confirmed that the Spanish version of the Lawton IADL Scale for older adults offers excellent reliability and validity, even though its sensitivity to change is only moderate.

When respondents were unable to perform one or more activities, they were considered to have a basic or instrumental disability.

#### Nutritional status

This was determined using the MNA Short Form (MNA-SF) [44]. This questionnaire is composed of six questions, with a maximum score of 14. A score of ≥ 11 indicates a risk of malnutrition.

#### Follow-up variables

The subjects were re-evaluated 2 years after the first interview (median 25 months). Previous falls were measured through the question: "*Have you had any falls in the last two years*?" An affirmative answer was considered to indicate that the subject had experienced at least a fall during the follow-up period. The number of falls experienced during the follow-up period was also noted.

### Ethical considerations

The study was approved by the Ethical Committee for Clinical Research of the *Arnau de Vilanova* University Hospital (S1 Report). Informed consent was obtained from all of the participants at the start of the interview.

### Statistical analyses

The statistical analysis included a descriptive analysis of the baseline characteristics of the sample. For the bivariate correlation analysis was used the Chi-square test. A multiple logistic regression analysis was subsequently performed. FOF was used as the outcome variable and significantly correlated factors were used as explanatory variables. Three models with increasing levels of adjustment were fitted: the first model contained crude estimates (model 1); age and gender were introduced in the second model (model 2); and health problems were incorporated into the third model (model 3) in order to obtain the final model.

Finally, falls at follow-up were modeled using the Cox proportional hazards regression. Three models, which included the same covariates described above, were presented (model 1–3). Model 3 additionally incorporated the covariate "previous falls". A *p* value of <0.05 was considered statistically significant. Statistical analysis was performed using the Statistical Package for Social Sciences (SPPS Inc, Chicago, IL) version 20.0.

## Results

A total of 640 subjects (60.3% female), with a mean age of 81.3 years old (SD 5.0), were included in the first wave of the study. Of these, 23.6% were 85 years old or older. In the following part of the study, the sample include 395 subjects. Thirty-three percent of our original sample were lost (5% due to death, 1% to institutionalization, 22.2% could not be located by telephone, and 5.1% refused to participate in the study).

The demographics and baseline characteristics are presented in Table 1. Half of the participants were married. In total, 74.2% of the participants had either started or completed primary education and 25% lived alone. Almost half of the participants reported having an annual income of less than €900. In the case of health problems, 36.5% presented low or high degrees of comorbidity, 7 out of 10 suffered moderate-to-severe cognitive status, and one quarter exhibited symptoms of depression. Some of the subjects presented either moderate (3%) or severe (5.5%) basic levels of disability, with instrumental disability being moderate in 21.6% of cases and severe in 13.8%. Finally, the participants presented a 21.3% risk of suffering malnutrition.

**Table 1:** Demographics and baseline characteristics: Number (n) and frequency (%).

Sociodemographic	n	%	Health status	n	%
Age*	81.5	(5.0)	Comorbidity		
Gender			Absence (0–1 points)	405	(63.3)
Men	254	(39.7)	Low (2 points)	102	(15.9)
Women	386	(60.3)	High (≥ 3 points)	132	(20.6)
Marital status			Cognitive status		
Single	24	(3.8)	Absence (0–2 errors)	533	(83.3)
Married	320	(50.0)	Mild (3–4 errors)	59	(9.2)
Living with a partner	3	(0.5)	Moderate (5–7 errors)	26	(4.1)
Separated	7	(1.1)	Severe (8–10 errors)	22	(3.4)
Widow/widower	286	(44.7)	Symptoms of depression		
Level of education			No	394	(74.3)
Illiterate	36	(5.6)	Yes	136	(25.7)
No education	198	(30.9)	Basic disability		
Primary (6–12 years old)	241	(37.7)	Absence (6 points)	497	(77.7)
Lower secondary (12–15 y.)	80	(12.5)	Mild (4–5 points)	88	(13.8)
Upper secondary (15–18 y.)	47	(7.3)	Moderate (2–3 points)	19	(3.0)
Higher education (≥ 18 y.)	38	(5.9)	Severe (0–1 points)	35	(5.5)
Living			Instrumental disability		
Alone	163	(25.5)	Absence (8 points)	412	(64.4)
Husband/wife	258	(40.3)	Moderate (4–7 points)	138	(21.6)
Children	105	(16.4)	Severe (0–3 points)	88	(13.8)
Husband/wife and children	47	(7.3)	Nutritional status		
Others	59	(9.2)	No risk of malnutrition	484	(78.7)
Income per month			Risk of malnutrition	131	(21.3)
< than €400	24	(3.8)	Fear of falling		
Between €400 and €600	161	(25.2)	No	337	(58.7)
Between €600 and €900	110	(17.2)	Yes	237	(41.3)
Between €900 and €1200	107	(16.7)			
Between €1200 and €1800	85	(13.3)			
> than €1800	45	(7.0)			
No answer	101	(15.8)			

*Mean and standard deviation (SD)

### Prevalence of falls and FOF

The prevalence of falls in the year previous to the study was 25% (95% CI 21.8–28.6), with 21.7% (95% CI 17.1–27.2) for men and 27.1% (95% CI 22.9–31.8) for women. 48.6% of the fallers had only suffered a single fall, as opposed to 52.4% who had suffered 2 falls or more.

The prevalence of FOF was 41.5% (95% CI 37.5–45.5), 21.8% (95% CI 16.9–27.4) in men and 55.0% in women (95% CI 49.7–60.2). In total, 36.5% of the subjects reporting FOF had experienced a fall in the previous 12 months.

### Falls at follow-up

The incidence of falls during the two-year follow-up period was 11.6% in men (95% CI 7.5–17.6) and 50.4% (95% CI 44.1–56.7) in women. 87.7% of the fallers suffered one fall, as opposed to 12.3% who suffered two or more falls.

### Fear of falling after a fall

Logistic regression models were constructed to analyze the potential relationship between FOF and a previous history of falls and sociodemographic and health-related variables (Table 2). Statistically significant differences were observed for the variable *previous falls* for all three models. Subjects who had experienced at least one previous fall were more likely to report FOF, even after adjusting for sociodemographic and health-related factors. The final model revealed that subjects with a previous history of falls were 2.5 times more likely to report FOF than those who had not suffered any falls in the previous year. The results were also significant for *gender*: women were found to be four times more likely to be afraid of falling than men and this significance persisted after adjusting for health-related factors in the final model. Significant differences were also observed in subjects with *comorbidities*, *symptoms of depression*, and *basic disability*, who were twice as likely to be afraid of falling than the other subjects. It is particularly interesting to note that none of the models showed *age* to have had any influence on FOF.

**Table 2:** Factors associated with a fear of falling based on having suffered previous falls. Univariate and multivariate analysis by logistic regression: Odds Ratio (OR) and 95% Confidence Interval (CI).

		Fear of falling						
			No (%)	Yes (%)	p	OR	CI 95% OR	p
Model 1	Previous falls	No	85.4	63.4				
		Yes	14.6	36.6	<0.001	3.19	2.10–4.85	< 0.001
Model 2	Previous falls	No	85.4	63.4				
		Yes	14.6	36.6	<0.001	3.23	2.06–5.05	< 0.001
	Gender	Male	54.6	21.1				
		Female	45.4	78.9	<0.001	4.33	2.87–6.54	< 0.001
	Age	< 85	81.9	75.9				
		≥ 85	18.1	24.1	0.08	1.43	0.89–2.30	0.14
Model 3	Previous falls	No	85.4	63.4				
		Yes	14.6	36.6	<0.001	2.48	1.54–3.97	< 0.001
	Gender (female)	Male	54.6	21.1				
		Female	45.4	78.9	<0.001	4.09	2.63–6.36	< 0.001
	Age (≥ 85 years old)	< 85	81.9	75.9				
		≥ 85	18.1	24.1	0.08	1.35	0.81–2.24	0.24
	Comorbidity	No	85.5	74.3				
		Yes	14.5	25.7	0.001	2.00	1.17–3.44	0.01
	Cognitive impairment	No	93.2	91.6				
		Yes	6.8	8.4	0.47	0.93	0.39–2.18	0.87
	Symptoms of depression	No	78.7	50.2				
		Yes	21.3	49.8	<0.001	2.51	1.63–3.89	< 0.001
	Disability	No	90.8	70.9				
		Yes	9.2	29.1	<0.001	2.56	1.45–4.50	0.001
	Risk of malnutrition	No	87.5	77.1				
		Yes	12.5	22.9	<0.001	1.20	0.69–2.08	0.50

### Fear of falling as a predictor of the risk of falling

In the Cox regression analysis (Table 3), the first model revealed that FOF was a predictor for the occurrence of falls reported at 25 months. In other words, subjects who were afraid of falling were twice as likely to experience a future fall than those who were not afraid of doing so. However, this association disappeared when sociodemographic variables were included in the second model. The final model showed that women were four times more likely to fall within a period of two years than men. Given the pronounced gender differences regarding the incidence of falls and FOF, we decided to analyze the HR estimates for FOF obtained for men and women. As shown in Table 4, none of the variables studied was predictive of falls in women; in the case of men, the only predictive variable was previous falls.

**Table 3:** Risk of suffering a fall during follow-up according to the fear of falling. Univariate and multivariate analysis by Cox regression analysis: Crude Hazard Ratio (CHR), Hazard Ratio (HR) and 95% Confidence Interval (CI).

		Fall during follow-up				CHR	95% CI	HR	95% CI	p
			No (%)	Yes (%)	p					
Model 1	Fear of falling	No	69.5	50.0						
		Yes	30.5	50.0	<0.001	1,74	1.22–2.48	1.93	1.33–2.81	0.001
Model 2	Fear of falling	No	69.5	50.0						
		Yes	30.5	50.0	<0.001	1,74	1.22–2.48	1.18	0.79–1.74	0.46
	Gender	Male	53.5	12.9						
		Female	46.5	87.1	<0.001	4,95	2.93–8.35	4.66	2.59–8.36	< 0.001
	Age	< 85	84.4	79.1						
		≥ 85	15.6	20.9	0.19	1,44	0.94–2.21	1.35	0.85–2.14	0.16
Model 3	Fear of falling	No	69.5	50.0						
		Yes	30.5	50.0	<0.001	1,74	1.22–2.48	1.18	0.77–1.81	0.43
	Gender	Male	53.5	12.9						
		Female	46.5	87.1	<0.001	4,95	2.93–8.35	4.42	2.44.7.99	< 0.001
	Age	< 85	84.4	79.1						
		≥ 85	15.6	20.9	0.19	1,44	0.94–2.21	1.49	0.92–2.40	0.09
	Comorbidity	No	79.3	78.4						
		Yes	20.7	21.6	0,04	1,03	0.66–1.58	1.02	0.60–1.75	0.92
	Cognitive impairment	No	86.7	87.8						
		Yes	13.3	12.2	0.76	0,87	0.51–1.47	0.45	0.18-1-12	0.08
	Symptoms of depression	No	71.7	65.0						
		Yes	28.3	35.0	0.20	1,36	0.93–1.99	1.05	0.67–1.64	0.81
	Disability	No	82.4	81.3						
		Yes	17.6	18.7	0.78	1,03	0.67–1.60	0.82	0.48–1.40	0.48
	Risk of malnutrition	No	88.4	76.3						
		Yes	11.6	23.7	0,02	2,68	1.16–1.72	1.39	0.81–2.38	0.23
	Previous falls	No	79.1	73.7						
		Yes	20.9	26.3	0.23	1,16	0.78–1.73	0.89	0.55–1.44	0.64

**Table 4:** Risk of suffering a fall during follow-up according to the fear of falling. Multivariate analysis by Cox regression analysis stratified by gender: Hazard Ratio (HR) and 95% Confidence Interval (CI).

	Woman				Men			
	HR	95% CI		p	HR	95% CI		p
		Lower	Upper			Lower	Upper	
Fear of falling	1.16	0.79	1.71	0.44	2.71	0.95	7.71	0.06
Age (≥ 85 years old)	0.89	0.56	1.41	0.62	1.60	0.52	4.96	0.41
Comorbidity	0.87	0.56	1.36	0.54	1.75	0.62	4.98	0.29
Cognitive impairment	0.91	0.51	1.64	0.76	0.42	0.06	3.19	0.40
Symptoms of depression	0.98	0.65	1.47	0.90	0.97	0.27	3.46	0.96
Disability	1.15	0.73	1.81	0.55	*			
Malnutrition risk	1.17	0.76	1.81	0.47	1.47	0.33	6.52	0.60
Previous falls	0.97	0.65	1.44	0.88	3.27	1.07	10.1	0.03

*None of the disabled men suffered falls according to the adjusted analysis; as a result, it was no possible to calculate the HR

## Discussion

### Prevalence of falls, fear of falling and falls during follow-up

The prevalence of previous falls obtained in this study was 25%. Similar prevalence rates had previously been reported in studies involving Spanish community-dwelling older adults aged over 70 [45,46]. In contrast, lower prevalence rates had been reported in other studies [47] involving subjects aged 65 to 75 years old. Given that the rate of falls tends to increase with age, this apparent inconsistency might have been related to the lower mean age of the sample. In studies conducted in other countries, similar prevalence rates have been reported [48,49].

After a two-year follow-up period, 35.2% of the study subjects had experienced at least one fall; this was consistent with the results of other previous longitudinal studies, which cited rates of suffering falls ranging from 29.1% to 42.2% [50]. In a systematic review of studies involving Chinese older adults, the annual prevalence rate of falls ranged from 11% to 34% in retrospective studies and from 15% to 16% in prospective studies [51]. The incidence of falls presented in the current study showed the same tendency; although if we compare this rate with the prevalence of falls in the initial evaluation, we observe that the total after two years was lower than expected. On explanation for this could be the difficulty remembering falls over longer periods of time. Another explanation could be the way in which the population was treated. While the initial evaluation was carried out at either the health center or the patient’s home, the follow up was carried out by telephone [52] and this could have influenced the results obtained [53].

The prevalence of FOF in our study was 41.5%. This rate was consistent with those reported in previous studies, in which it ranged from 20 to 60% [4,8,10,19,54–57].

Despite the variability in the measurement tools used and differences in the ages of those studied, the prevalence rates were consistent with those obtained in the most recent studies.

### Fear of falling as a cause and/or consequence of falls

This study supports the widely accepted impression that a previous history of falls is a risk factor for FOF [6,19,56]. The subjects who had suffered a previous fall were twice as likely to be afraid of falling than those who had not experienced any (OR 2.48), even after adjusting for sociodemographic factors and health status.

As well as previous falls, the final logistic regression model revealed that female gender, comorbidities, symptoms of depression, and disability were the main factors associated with FOF; this confirmed the multifactorial construct of this geriatric syndrome. This was consistent with the results of previous studies that associated the FOF with previous falls, female gender, chronic disease, symptoms of depression and limitations in ADL [58,59]. These studies also associated being > 80 years old and having a FOF. However, this finding was not supported by the results of our study, as well as in another recent one [60]. One possible explanation for this would be the increase in the prevalence of cognitive impairment with advancing age. Individuals with > 3 errors on the Pfeiffer test were excluded from the study and this may have limited our ability to demonstrate the relationship between age and FOF.

On the other hand, the crude absolute risks for subjects who fell with FOF was 31.2%, and of those without FOF was 19.2%. There is also evidence that FOF can be used to predict falls [12,23,58]. The results of the present study support this conclusion, although the single-item question predicted falls only using the unadjusted model (HR 1.93; CI 95%: 1.33–2.81; p = 0.001) and the differences were not significant after adjustment. Other studies have reported similar results [23,26]. These contradict those presented by Friedman et al. [12], who identified white race, female gender, a history of strokes, sedative use and FOF at baseline as independent predictors for new incidences of falling.

In fact, the only variable that was found to be a determinant of the occurrence of falls after two years was female gender [12,58]: women were 4.5 times more likely to experience a fall after two years than men.

Given the pronounced gender differences that were observed in our study with regard to the incidence of suffering falls and FOF, we applied a Cox model to analyze the HR for men and for women. According to these results, there was a significant interaction with sex. In particular, there was a different relationship with respect to using the history of previous falls to predict falls in follow-up, since this factor was only associated for men. Special attention should be paid to the long-term consequences of FOF, since it can lead to a poorer health status, cause functional decline, and result in a loss of autonomy [55]. These adverse outcomes support recommendations for older adults to be considered a risk population.

The results obtained show that this vulnerable group needs to receive integrated care. It is therefore important that family and community nurses appropriately assess the risk of suffering falls and FOF in older adults in their care and implement effective preventive strategies. Such interventions could help to limit and prevent both FOF and repeated falls, as these two problems are interrelated.

### Study limitations

Our study had several limitations. First, we used only a single question ("*Are you afraid of falling*?") to measure the outcome. Dichotomous questions have been used by different authors [61,62] and their reliability and validity have been amply demonstrated [63]. Perhaps, this short question should have been accompanied by an explanation from the interviewer of what exactly they wanted to establish. FOF may be attributable to a general feeling of anxiety associated with many different factors, including falling and losing balance, or it may be induced by the restriction of a particular activity. This could explain why 10% of the respondents either did not answer the question or stated that they did not know what to answer when they were interviewed. The approach of using a single question to assess FOF has previously been shown to be less sensitive than other broader measures such as the Falls Efficacy Scale (FES) [20,64,65]; even so, this dichotomous question has been shown to provide an acceptable level of predictive validity and a notably high point-biserial correlation with the full FES-I; as such, it could therefore be judged relevant for future clinical use [63]. Another limitation in this study was related to the fact that, when asked about their history of previous falls, some subjects may not have remembered recent falls; this theory is supported by a HR of less than 0.5 in the Cox model 3 (although this was not statistically significant). It is for this reason that it was decided that if there were 3 or more errors in the Pfeiffer test the question had to be answered by a PROXY. Finally, FOF was not reevaluated at follow-up because we thought that this was not a variable that should be used in the current study. In any new research, it would be interesting to have this information and to know how well FOF is predicted based on previous falls.

## Conclusion

The main contribution of this study to the field of community nursing is that it demonstrates that previous falls and comorbidities are associated with FOF and confirms the multifactorial etiology of this psychological phenomenon. Evidence is also provided to show that FOF was a predictor of falls during follow-up only in the unadjusted model, pointing to strong gender turns out as an effect modifier of the association of FOF and subsequent falls. It is important that nurses appropriately assess the risk of falls and FOF in older adults in their care and implement effective preventive strategies in the community.

## Supporting information

S1 Dataset and Metafile (XLSX)

S1 Report From ethics committee for clinical research. (PDF)
