# Correction: Fear of falling in community-dwelling older adults: A cause of falls, a consequence, or both?

**DOI:** 10.1371/journal.pone.0197792

**Published:** 2018-05-17

**Authors:** Ana Lavedán, Maria Viladrosa, Pilar Jürschik, Teresa Botigué, Carmen Nuín, Olga Masot, Raquel Lavedán

There are errors in the author affiliations. The affiliations should appear as shown here:

Ana Lavedán^1,4^, Maria Viladrosa^1,2,4^, Pilar Jürschik^1,4^, Teresa Botigué^1,4^, Carmen Nuín^1,4^, Olga Masot^1,4^, Raquel Lavedán^3^

**1** Department of Nursing and Physiotherapy, University of Lleida, Lleida, Spain, **2** University Hospital Arnau de Vilanova, Lleida, Spain, **3** University Clinical Hospital Lozano Blesa, Zaragoza, Spain, **4** Health Care Research Group (GRECS), Biomedical Research Institute of Lleida, Spain.

## References

[1] Lavedán A, Viladrosa M, Jürschik P, Botigué T, Nuín C, Masot O, et al (2018) Fear of falling in community-dwelling older adults: A cause of falls, a consequence, or both? PLoS ONE 13(3): e0194967 https://doi.org/10.1371/journal.pone.0194967 29596521 10.1371/journal.pone.0194967 PMC5875785

