# Supplementary material for: Fear of falling in community-dwelling older adults: A cause of falls, a consequence, or both?
Source: PLoS One. 2018 Mar 29;13(3):e0194967. doi: 10.1371/journal.pone.0194967 (PMC5875785; doi:10.1371/journal.pone.0194967)
Supplement: S1 Report — (PDF) [file pone.0194967.s002.pdf]

Institut Català de la Salut
Lleida
Hospital Universitari Arnau de Vilanova

El Comité Ético de Investigación Clínica en la reunión de 30 de octubre de 2008, acta 13/2008, informó favorablemente la solicitud del proyecto de investigación titulado: **"Evolución del proceso de fragilidad de la población mayor de Lleida"** con la Dra. Pilar Jürschik Jiménez como investigadora principal en la Escuela Universitaria de Enfermería de la Universitat de Lleida, y consideró que:

- Se cumplen los requisitos necesarios de idoneidad del protocolo en relación a los objetivos del estudio y que estan justificados los riesgos y molestias previsibles para los sujetos participantes.

- La capacidad del investigador y los medios de que dispone son apropiados para llevar a cabo el estudio.

- Es adecuado el procedimiento para obtener el consentimiento informado de los sujetos que participan en el estudio.

Lleida, 27 de noviembre de 2008

Joan Antoni Schoenenberger Arnaiz
Presidente
